# Deceleration during 'real life' motor vehicle collisions – a sensitive predictor for the risk of sustaining a cervical spine injury?

**DOI:** 10.1186/1754-9493-3-5

**Published:** 2009-03-08

**Authors:** Martin Elbel, Michael Kramer, Markus Huber-Lang, Erich Hartwig, Christoph Dehner

**Affiliations:** 1 Center of Surgery, Department of Orthopedic Trauma, Hand, Plastic and Reconstructive Surgery, University of Ulm, Steinhövelstrasse 9, 89075, Ulm, Germany; 2 Department of Trauma Surgery, Deaconesses Hospital, Karlsruhe – Rüppurr, Academic Teaching Hospital of Freiburg University, Diakonissenstrasse 28, 76199, Karlsruhe, Germany

## Abstract

**Background:**

The predictive value of trauma impact for the severity of whiplash injuries has mainly been investigated in sled- and crash-test studies. However, very little data exist for real-life accidents. Therefore, the predictive value of the trauma impact as assessed by the change in velocity of the car due to the collision (ΔV) for the resulting cervical spine injuries were investigated in 57 cases after real-life car accidents.

**Methods:**

ΔV was determined for every car and clinical findings related to the cervical spine were assessed and classified according to the Quebec Task Force (QTF).

**Results:**

In our study, 32 (56%) subjects did not complain about symptoms and were therefore classified as QTF grade 0; 25 (44%) patients complained of neck pain: 8 (14%) were classified as QTF grade I, 6 (10%) as QTF grade II, and 11 (19%) as QTF grade IV. Only a slight correlation (r = 0.55) was found between the reported pain and ΔV. No relevant correlation was found between ΔV and the neck disability index (r = 0.46) and between ΔV and the QTF grade (r = 0.45) for any of the collision types. There was no ΔV threshold associated with acceptable sensitivity and specificity for the prognosis of a cervical spine injury.

**Conclusion:**

The results of this study indicate that ΔV is not a conclusive predictor for cervical spine injury in real-life motor vehicle accidents. This is of importance for surgeons involved in medicolegal expertise jobs as well as patients who suffer from whiplash-associated disorders (WADs) after motor vehicle accidents.

**Trial registration:**

The study complied with applicable German law and with the principles of the Helsinki Declaration and was approved by the institutional ethics commission.

## Background

Whiplash injuries remain a barely understood phenomenon. The economic damage caused by whiplash amounts to some 10 billion Euros a year in Europe [1] and 29 billion US Dollars a year in the USA [2]. As whiplash occurs as a result of motor vehicle accidents (MVAs), questions inevitably arise regarding who is liable for these costs.

Biomechanical considerations have been based on the assumption that damage to a given material only occurs when the energy that acts on this material is high enough. Thus, energy doses below a defined threshold have been considered harmless [3,4]. In this context, the parameter delta v (ΔV), which describes the velocity change of a motor vehicle during a collision with another vehicle, has become a widely accepted criterion for the energy that acts on the vehicle during a collision [5].

In numerous sled or car crash-test studies, volunteers were subjected to acceleration forces in order to define a threshold below which a cervical spine injury could be excluded [6-15]. The results of these studies are rather inconclusive and sometimes contradictory. Thus the scientific community has not yet reached consensus regarding the threshold value for cervical spine injuries after whiplash. Nonetheless, ΔV threshold values were adopted very early in the history of insurance law as a criterion to accept or deny the claim settlement for whiplash-associated disorders (WADs) [16].

Up until now, all volunteer crash-test studies precisely defined the subject's sitting position. While waiting for the collision, the subjects maintained an upright body and head position, with an optimally adjusted headrest. It is obvious that the real-life sitting position in traffic may significantly differ from this laboratory position in one or several points. Furthermore, an increased risk of injury has been observed for various factors such as the seat and headrest settings [11,17-20], the distance between head and headrest [21-23], the head rotation, and the collision type [24]. The inherent variability of these factors makes it unclear how easily the results from laboratory crash tests can be transferred to real-life accident situations. In order to elucidate these issues, this study analyzes the correlation between ΔV and cervical spine injuries in real-life accidents and questions whether ΔV is a valid predictor for cervical spine injuries following whiplash.

## Methods

The study included 57 patients after a car collision. The patients were recruited either by an engineer's office for vehicle damage assessment and claims adjustment (n = 46) or by the first consultation of an emergency room (n = 11). We obtained the approval of the local independent ethics board and all patients gave their written informed consent to participate in the study.

### Clinical Data

The clinical data were collected within 48 h after occurrence of the accident. Neck pain was determined on a visual analog scale (VAS) ranging from 0 (no pain) to 100 (maximal pain). The neck disability index (NDI) was used to assess disability problems related to neck pain. The NDI includes 10 items that attempt to describe the impact of neck pain: pain intensity, personal care, lifting, reading, headaches, concentration, work, driving, sleeping and recreation [25]. Subjects are requested to choose for each item, the statement that best describes their current situation; the statements represent different grades of severity. A total score which ranges from 0 to 50 was finally derived as the sum of the ten items.

All subjects who reported neck pain were physically and radiologically examined. The physical examination included investigation of the cranial nerves as well as of the motor and sensory function of spinal nerves C5–C8. Areas that were painful upon application of pressure were also examined. Furthermore, the range of motion (ROM) of the cervical spine in flexion/extension, rotation and lateral flexion was measured. In addition, X-rays of the cervical spine were taken in two planes. A CT scan was additionally taken if pathological findings were noted. The clinical and radiological findings were used to classify the whiplash injury according to the Quebec Task Force (QTF) system [26] (Table 1). The medical investigator was blinded concerning the technical data. Patients were informed of all results from the clinical examination excluding the QTF values.

**Table 1:** Clinical classification of whiplash-associated disorders according to the Quebec Task Force

**QTF Grade**	**Clinical Symptoms**
**0**	**No complaint **about the neck, no physical signs
**I**	Neck complaint of pain, stiffness or tenderness only, **no physical signs**
**II**	Neck complaint and musculoskeletal signs *
**III**	Neck complaint and neurological signs **
**IV**	Neck complaint and **fracture or dislocation**

* Musculoskeletal signs include decreased range of motion and point tenderness; ** Neurologic signs include decreased or absent deep tendon reflexes, weakness and sensory deficits; Symptoms and disorders that can be manifest in all grades include deafness, dizziness, tinnitus, headache, memory loss, dysphagia and temporomandibular joint pain

### Technical Data

In addition to the clinical findings, the ΔVs of their respective accident vehicles were determined for all patients. The damage on all vehicles involved in the accidents was examined by a certified engineer who was experienced in the assessment of such damage. The ΔV and the collision type (frontal, rear-end, side collision, multiple collisions, rollovers) were determined on the basis of the damage sustained by the vehicles. Depending on the available data, the ΔV was analyzed either by calculation and graphic illustration [27] or with the EES method [28]. The engineer was blinded concerning the clinical examination results.

### Statistics

Descriptive analysis was performed for all parameters. Pearson's correlation coefficient was determined for the correlation between the pain score (VAS) and ΔV and for the correlation between the NDI and ΔV. The correlation between QTF classification and ΔV was described by Spearman's correlation coefficient. The specificity and sensitivity were calculated for the hypothesis that no cervical spine injuries occur below a particular ΔV threshold and that injuries can occur above this threshold. P-values below 0.05 were considered significant.

## Results

We enrolled 57 individuals (25 males and 32 females) in the study; these individuals had been the occupants of 51 cars (Table 2). The median age was 33 (range 3 to 90 years) for the males and 30 (range 18 to 59 years) for the females.

**Table 2:** Collision type, delta V, sex, age, QTF grade, pain score, neck disability index (NDI) and description of injury in cases of QTF grade IV in all studied subjects.

**No**.	**Collision**	**Delta V**	**Sex**	**Age**	**QTF**	**Pain score**	**NDI**	**Injury**
1	Frontal	8	♂	30	0	0	0	
2	Frontal	11	♂	19	0	0	0	
3	Frontal	15	♂	21	0	0	0	
4	Frontal	16	♀	33	0	0	0	
5	Frontal	17	♂	38	0	0	0	
6	Frontal	17	♂	24	0	0	0	
7	Frontal	24	♂	56	0	0	0	
8	Frontal	25	♀	20	0	0	0	
9	Frontal	28	♂	-	0	0	0	
10	Frontal	18	♀	26	1	49	17	
11	Frontal	15	♀	37	4	91	44	Fracture at C7 with dislocation at C6/7
12	Frontal	32	♀	20	4	89	40	Fracture at C5 with dislocation at C5/6, paraplegia at C7
13	Frontal	50	♂	20	4	89	36	Fracture at C5 with dislocation at C4/5
14	Rear	3	♀	39	0	0	0	
15	Rear	6	♀	40	0	0	0	
16	Rear	8	♂	-	0	0	0	
17	Rear	9	♀	27	0	0	0	
18	Rear	9	♂	20	0	0	0	
19	Rear	11	♂	23	0	0	0	
20	Rear	11	♀	31	0	0	0	
21	Rear	12	♀	31	0	0	0	
22	Rear	13	♀	36	0	0	0	
23	Rear	15	♀	59	0	0	0	
24	Rear	15	♂	27	0	0	0	
25	Rear	24	♂	53	0	0	0	
26	Rear	37	♂	42	0	0	0	
27	Rear	9	♂	31	1	70	10	
28	Rear	9	♀	26	1	51	18	
29	Rear	11	♀	31	1	36	4	
30	Rear	23	♀	19	1	50	5	
31	Rear	24	♀	58	1	44	17	
32	Rear	58	♀	19	1	26	8	
33	Rear	15	♀	23	2	49	14	
34	Rear	20	♀	31	2	55	16	
35	Side	4	♀	23	0	0	0	
36	Side	7	♀	39	0	0	0	
37	Side	9	♂	34	0	0	0	
38	Side	10	♂	54	0	0	0	
39	Side	10	♀	30	0	0	0	
40	Side	10	♂	51	0	0	0	
41	Side	11	♂	56	0	0	0	
42	Side	14	♂	42	0	0	0	
43	Side	18	♀	22	0	0	0	
44	Side	36	♀	29	0	0	0	
45	Side	9	♀	55	2	7	16	
46	Side	10	♀	59	2	61	20	
47	Side	10	♂	33	4	83	35	Zygapophyseal joint fracture at C4
48	Side	16	♂	90	4	94	46	Fracture at C7 with dislocation at C6/7
49	Side	32	♀	29	4	86	33	Zygapophyseal joint fracture at C2
50	Side	50	♂	33	4	81	47	Fracture at C5 with dislocation at C5/6, paraplegia at C6
51	Side	52	♀	34	4	92	34	Bony rupture of the alar ligaments
52	Side	58	♂	3	4	96	49	Atlantoaxial dislocation
53	Side	59	♀	39	4	87	48	Bony rupture of the alar ligaments
54	Multiple	46	♀	18	1	69	21	
55	Multiple	33	♀	32	2	67	38	
56	Multiple	46	♀	19	2	85	24	
57	Rollover	15	♀	30	4	94	42	Dens axis fracture Anderson 2

**Figure 1:**
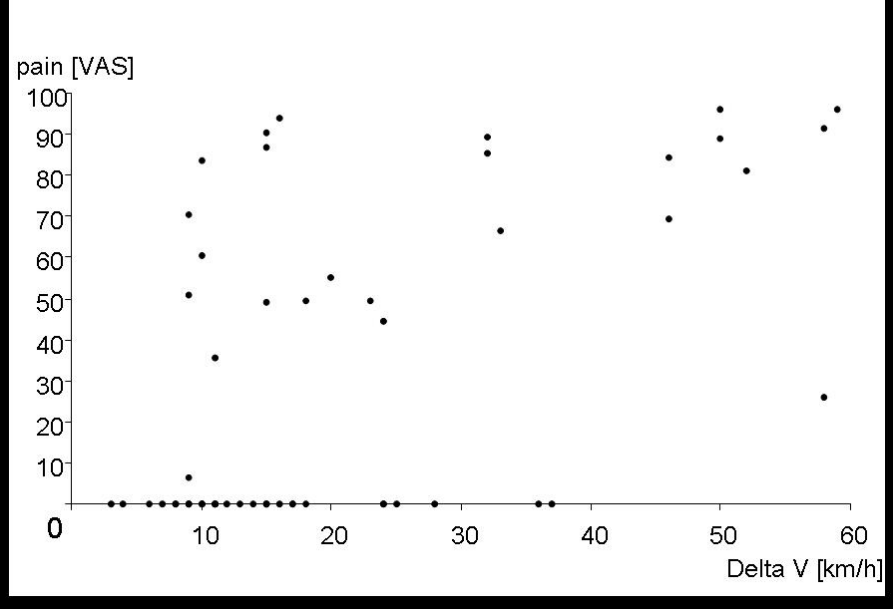
**Pain score (VAS) for all subjects (n = 57) as a function of delta V (km/h)**.

A total of 25 (44%) patients complained about pain in the neck. VAS pain scores of 7 to 96 (median = 71) were reported. Fifteen patients reported an immediate onset of pain, four individuals reported a time to onset of minutes to hours, and four patients reported a time to onset of hours to one day. The Pearson's correlation coefficient of r = 0.55 indicated a moderate correlation between the pain that was subjectively reported and ΔV (Fig. 1).

**Figure 2:**
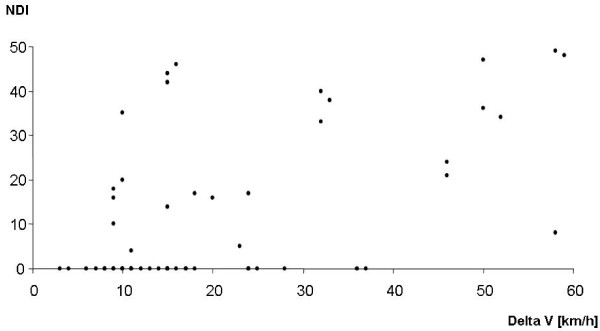
**Neck disability index (NDI) for all subjects (n = 57) as a function of delta V (km/h)**.

A total of 25 (44%) patients complained pain related neck disability. NDI scores of 4 to 49 (median = 24) were reported. The Pearson's correlation coefficient of r = 0.46 indicated no relevant correlation between the NDI and ΔV (Fig. 2).

**Figure 3:**
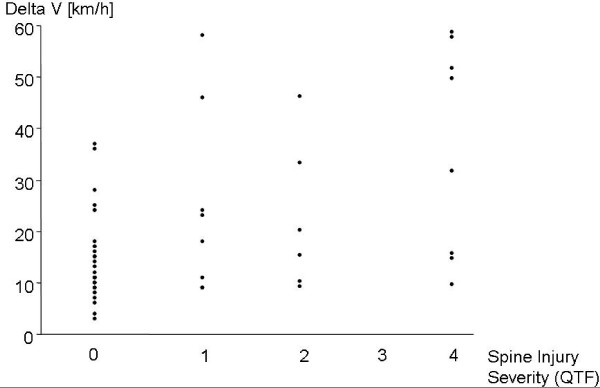
**Delta V for all subjects (n = 57) as a function of the spine injury severity (QTF grade) (QTF 0: n = 32, QTF I: n = 8, QTF II: n = 6, QTF IV: n = 11)**.

Thirty-two patients (56%) were classified as QTF grade 0. Eight patients (14%) presented with QTF grade I, 6 patients (10%) with QTF grade II, and 11 patients (19%) with QTF grade IV. No QTF grade III injuries were scored. The Spearman's correlation coefficient of r = 0.45 indicated no relevant correlation between ΔV and the QTF grade of cervical spine injury (Fig. 3).

Rear-end collision (n = 21, 36%) was the most frequent collision type, followed by side collisions (n = 19, 33%) and front collisions (n = 13, 23%); there were also three multiple collisions and one rollover. For the rear-end collisions, individuals with and without cervical spine injuries were found in a ΔV range between 9 km/h and 37 km/h. This range was 15 km/h to 28 km/h for frontal collisions and 9 km/h to 36 km/h for side collisions. Within these ranges, the percentage of false-positive and false-negative results varied greatly, depending on the predefined cut-off values (Tables 3, 4 and 5). Therefore, for all collision types it was impossible to define a ΔV value that excluded the occurrence of cervical spine injury with acceptable sensitivity while simultaneously predicting the occurrence of cervical spine injury with acceptable specificity.

**Table 3:** Specificity and sensitivity for specific delta V threshold values in frontal collisions (n = 13).

**Delta V [km/h]**	**Sensitivity**	**Specificity**
8	0%	100%
15	33%	75%
20	67%	50%
35	100%	25%

**Table 4:** Specificity and sensitivity for specific delta V threshold values in rear-end collisions (n = 21).

**Delta V [km/h]**	**Sensitivity**	**Specificity**
4	0%	100%
10	38%	75%
15	85%	50%
25	92%	13%
40	100%	13%

**Table 5:** Specificity and sensitivity for specific delta V threshold values in side collisions (n = 19).

**Delta V [km/h]**	**Sensitivity**	**Specificity**
4	0%	100%
10	60%	67%
20	90%	56%
35	100%	44%
60	100%	0%

## Discussion

This study provides evidence that, in real-life accidents, cervical spine injuries may occur at low ΔV values, while it is possible to escape unscathed from collisions with high ΔV values. In particular, the correlation between ΔV and the occurrence of WADs was very low for any of the collision types. Therefore it is impossible to make meaningful statements about the existence of WAD based solely on assessment of the ΔV value. This finding might be of importance for the surgeon's assessment and patient's safety after a car accident. Diagnostic and therapeutic management should not be based solely on information related to trauma impact.

The results of the present study support the findings of numerous sled and car-crash experiments. In those experiments, neck problems were noted after rear-end collisions with ΔVs as low as 7 km/h [14,29-31]. In four other studies [8,32-34], neck problems occurred at a ΔV < 10 km/h. The neck problems were defined as QTF grade I and QTF grade II, persisting from hours to several weeks in all studies. In contrast, four studies reported rear-end collisions with ΔV values of 13.1 km/h to 50 km/h where the occupants escaped without any signs of injury [4,9,35,36]. In other crash-test studies, frontal impacts at ΔV less than 12 km/h caused no injuries [34]. However, different findings were obtained in our study and in a study that performed a retrospective analysis of 24 real-life frontal collisions [37]. In that study, 18 of the 24 subjects were classified as QTF grade II. It is noteworthy that 8 of these had neck problems for more than one year. The ΔVs in these cases ranged from 3 km/h to 23 km/h. The authors also reported that one subject suffered a prolapsed disk at C5/6 at a ΔV of 11–15 km/h. The occupant had not been wearing his seat belt and the airbag had deployed. He also had a frontal laceration as a sign of direct head impact. It was assumed that these factors caused the structural injury of the cervical spine at a low ΔV. The occurrence of structural injuries at ΔV values of less than 20 km/h had been considered improbable in expert discussions. However, we also observed a luxation fracture at C5/6 resulting from a frontal collision at a ΔV of 15 km/h and a facet joint fracture at C4 due to a side collision at a ΔV of 10 km/h (Table 1). Both occupants had been wearing their seat belts, there had been no head contact, and the airbag had not deployed. In both cases, it is unclear which factors, either alone or in combination, were responsible for these structural injuries at considerably low ΔV. In accordance with other studies mentioned below, these results are indicative that multiple factors may influence the risk of injury in each individual case. Due to the additive effects of various protective factors, high-energy impacts may be absorbed without injury, while the additive effects of unfavorable factors could explain injuries sustained in low-energy impacts. Some factors have been described to influence the risk of injury, such as sex [38,39], head position [40], sitting position [24,41], distance between head and headrest [21-23] and seat construction [11,17-19]. The duration of the crash pulse is also thought to significantly contribute to the risk of cervical spine injury. These authors stated that an earlier acceleration peak during deformation of the colliding cars was correlated with a higher probability of cervical spine injury [24]. However, it remains unclear to what extent each one of these factors influences the risk of cervical spine injury.

The current data exclude the assumption of a linear correlation between ΔV and the risk of suffering a whiplash injury. It is tempting to speculate that the development of a cervical spine injury after whiplash is more like a complex system such as those described in chaos theory [42]. Complex systems cannot be simplified into linear correlations. Even small variations of the initial conditions can affect the end result so that it is no longer predictable, such as in the case of the "butterfly effect": the flapping of a butterfly's wings can ultimately result in a different weather pattern [43]. Taken together, it can be concluded that ΔV is an irrelevant predictive value for cervical spine injury after a MVA. Nevertheless further studies will be necessary to evaluate the development of pain chronification in dependence of the ΔV to investigate its possible predictive value as "long-term" parameter.

## Conclusion

The ΔV value as measured in the trauma impact does not represent a conclusive predictor for cervical spine injury in real-life motor vehicle accidents. This could be important for surgeons and patients in their medicolegal assessment of WADs.

## Competing interests

The authors declare that they have no competing interests.

## Authors' contributions

ME drafted the manuscript and performed the medical examination. MK participated in the study coordination and helped in the medical examination. MHL helped to draft the manuscript. EH participated in the study design and its coordination. CD performed the statistical analysis and helped to draft the manuscript. All authors read and approved the final manuscript.
